# Evaluation of Preemergent Herbicides for *Chloris virgata* Control in Mungbean

**DOI:** 10.3390/plants10081632

**Published:** 2021-08-09

**Authors:** Gulshan Mahajan, Bhagirath S. Chauhan

**Affiliations:** 1 Queensland Alliance for Agriculture and Food Innovation (QAAFI), The University of Queensland, Gatton, QLD 4343, Australia; 2 Department of Agronomy, Punjab Agricultural University, Ludhiana 141004, India; 3 Queensland Alliance for Agriculture and Food Innovation (QAAFI) and School of Agriculture and Food Sciences (SAFS), The University of Queensland, Gatton, QLD 4343, Australia; b.chauhan@uq.edu.au; 4 Department of Agronomy, Chaudhary Charan Singh Haryana Agricultural University (CCSHAU), Hisar 125004, India

**Keywords:** crop-toxicity, feather fingergrass, herbicide dose, pulses, seed number, weed biomass

## Abstract

*Chloris virgata* is a problematic weed in mungbean crops due to its high seed production, resistance to glyphosate and high dispersal ability. Pot and field experiments were conducted in 2020 and 2021 to evaluate a range of preemergent (PRE) herbicides for *C. virgata* control in mungbean. In the field and pot studies, isoxaflutole 75 g ai ha^−1^ caused crop injury, and in the field experiment, it reduced mungbean yield by 61% compared with the best treatment (pyroxasulfone 100 g ai ha^−1^). In the field and pot experiments, dimethenamid-P 720 g ai ha^−1^, pyroxasulfone 100 g ai ha^−1^ and S-metolachlor 1400 g ai ha^−1^ provided >88% control of *C. virgata* (for reduced biomass) and in the field experiment, these herbicides resulted in improved yield by 230%, 270% and 170%, respectively, compared with nontreated control (250 kg ha^−1^). Similarly, pendimethalin 1000 g ai ha^−1^ and trifluralin 600 g ai ha^−1^ provided >89% control (biomass) of *C. virgata,* and in the field experiment, these resulted in improved yields of 230% and 160%, respectively, compared with the nontreated control. PRE herbicides such as diuron 750 g ai ha^−1^, linuron 1100 g ai ha^−1^, metribuzin 360 g ha^−1^, terbuthylazine 750 g ai ha^−1^, imazapic 48 g ai ha^−1^ and imazethapyr 70 g ha^−1^ although did not cause crop injury; however, these herbicides did not control *C. virgata*. Flumioxazin 90 g ai ha^−1^ caused reduced biomass of *C. virgata* by 80% compared with the nontreated control, and in the field experiment, it resulted in improved yield by 140% compared with the nontreated control. This study suggests the potential use of herbicides, such as dimethenamid-P, pyroxasulfone and S-metolachlor in addition to pendimethalin and trifluralin, for *C. virgata* control in mungbean. Further studies are needed to determine the efficacy of dimethenamid-P, S-metolachlor and pyroxasulfone for controlling other troublesome weeds in mungbean.

## 1. Introduction

Mungbean [*Vigna radiata* (L.) R. Wilczek] is an important export potential crop of Australia. During 2015–2016, mungbean was planted in 1.25 m ha and its export was worth AUD 180 million [1,2]. Weed management in mungbean is one of the main production constraints for growers as mungbean plants are short stature, grow slowly and therefore, are poor competitors with weeds. Previous studies revealed that weed interference in mungbean can reduce its grain yield by as much as 87% [3,4]. Weed infestation can also reduce the grain quality of mungbean. 

*Chloris virgata* Sw.is a problematic warm-season weed of eastern Australia and its occurrence can also be seen throughout the Australian mainland [5,6,7]. High seed production, dispersal through wind and flood water, and tolerance or resistance to glyphosate have resulted in the prevalence *C. virgata* in both cropping and non-cropping areas in Australia [8,9]. Recently, it was estimated that *C. virgata* caused a total grain loss of 0.4 mt annually, resulting in a revenue loss of AUD 7.7 million [10]. *C. virgata* is a fast-growing weed with high biomass production and one plant can have a biomass 700 g m^−2^ [11]. In mungbean, about 50 *C. virgata* plants m^−2^ caused a yield loss of greater than 70% [12]. At the same infestation level, *C. virgata* produced about 370,000 seeds m^–2^. These observations suggest that with a lack of proper weed management, *C. virgata* can proliferate in mungbean paddocks, leading to huge yield losses in cropping land.

In a survey report of 2008 and 2010, *C. virgata* was ranked in the top 20 weeds of Australia [13]. A recent survey in the cotton-growing regions of Australia ranked *C. virgata* as the sixth most common weed species [14]. It was opined that *C. virgata* became tolerant to glyphosate due to overreliance on this herbicide in the no-till system. 

ACCase-inhibitor herbicides are used for *C. virgata* control; however, recent field observations suggest that *C. virgata* has evolved resistance to some of these herbicides in some paddocks [15]. Management of glyphosate-resistant and ACCase-inhibitor herbicide-resistant weeds requires the use of preemergent (PRE) herbicides that may prove effective on *C. virgata* as a part of the weed management system.

Poor control of *C. virgata* has been identified as a major cause of low yields in mungbean [12,15]. Although competition from weeds occurs at all periods of growth, the most damaging effects of *C. virgata* on mungbean occur when the crop is in its early canopy-formation stage [12]. Several studies have emphasized the importance of early weed control in mungbean to achieve high yields [16,17,18].

There are a limited number of PRE herbicides available for *C. virgata* control in mungbean. The widely used PRE herbicides currently used by mungbean growers in Australia are pendimethalin and trifluralin; however, the efficacy of these herbicides may vary with different soil textures and moisture [15]. It is therefore important to provide farmers with efficient and sustainable weed management packages to enhance mungbean yields. Efficient weed control in mungbean may vary with the type of herbicide used, soil type and weed flora present in the field. Research is needed to identify PRE herbicides that could provide effective control of *C. virgata* in mungbean. Therefore, field and pot studies were conducted to evaluate the performance of different PRE herbicides on *C. virgata* and mungbean toxicity and grain yield. The novelty of this research is to find an alternative of pendimethalin and trifluralin in the PRE herbicide program for more flexibility in herbicide options for *C. virgata* control in mungbean and delaying the evolution of herbicide resistance.

## 2. Materials and Methods

### 2.1. Experiment 1. Field Study

A field experiment was conducted in the spring and summer seasons of 2020–2021 at the Gatton Research Farm (27.5514° S and 152.3428° E) of the University of Queensland, Australia, to evaluate PRE herbicides (Tables 1 and 2) for the control of *C. virgata* in mungbean. The experiment was conducted in a randomized complete block design with three replications. The plot size was 6.0 by 1.4 m. The soil type at the experimental site was medium clay with 1.3% organic matter and a pH of 6.9. The mungbean variety Jade Au was used and the crop was planted on 22 September 2020 and harvested on 22 January 2021.

Before mungbean planting, the field was cultivated two times with a rotary cultivator to ensure a fine seedbed. After tillage, the field was infested with *C. virgata* seeds. For this purpose, *C. virgata* seeds (approximately 500 g) were mixed with sand and broadcasted uniformly over the plots. After weed infestation and prior to sowing, PRE herbicides were applied with a water volume of 160 L ha^−1^ through a CO_2_-pressurised backpack sprayer by maintaining a pressure of 200 kPa. The sprayer had four flat-fan nozzles (AIRMIX 110,015 Quick TeeJet nozzles, Model 25611) that were fixed at 50 cm spacing.

After herbicide application, mungbean was sown at a seeding rate of 30 kg ha^−1^ at 35-cm row spacing using a cone planter. The planter had a low-soil disturbance disc system and therefore, there was limited incorporation of PRE herbicides by the sowing operation. The field was surface irrigated immediately after sowing using a sprinkler system. 

*Chloris virgata* density, seed head and biomass were determined at the maturity stage of the crop by using a quadrat (50 cm × 50 cm) placed randomly at two places in each plot. Weeds in each plot were counted and samples were collected by cutting *C. virgata* plants at the base level and samples were dried in an oven at 70 C for 72 h. To estimate seed numbers of *C. virgata*, lobes in each seed head and rachilla segment (pedicel base) in each lobe were counted [19].

The crop was harvested using a combine harvester and grain yield was recorded from a net plot area of 7.0 m^2^ (5 m × 1.4 m) per plot. For ease of harvesting, the mungbean crop was desiccated in the last week of December [at the physiological maturity stage of the crop using a foliar spray of glyphosate (1900 g a.e. ha^−1^) plus saflufenacil (24 g ai ha^−1^)]. Grain yield was converted to kg ha^−1^ at a 12% moisture content.

### 2.2. Experiment 2. Pot Study (PRE Herbicides)

This pot experiment was started on 22 October 2020 using treatments listed in Table 3 to further confirm the efficacy of herbicides against *C. virgata* that were tested under field conditions. The experiment was repeated once with the same treatments on 22 February 2021. In this case, 20 seeds each of *C. virgata* and mungbean were sown separately in each pot (20 cm diameter and 18 cm height) filled with potting mix (Centenary Landscape, Australia). Pots were placed outside on benches and arranged in a randomized complete block design replicated thrice. 

Herbicide spray was carried out immediately after sowing and after spraying, pots were covered with a thin layer (5 mm) of potting mix. Herbicide spray was carried out using a research track sprayer. Pots were treated with herbicides as per treatment using a spray volume of 108 L ha^−1^ and Teejet XR 110,015 flat fan nozzles were used. Pots were kept without watering for 24 h after spray, and thereafter, pots were regularly irrigated using an automated sprinkler system. 

Plant survival was assessed at 28 days after treatment (DAT), and aboveground biomass was harvested, dried for 72 h at 70 °C and weighed. 

### 2.3. Experiment 3. Pot Study (Imazapic Doses)

In Australia, imazapic is being used for *C. virgata* control as a residual herbicide (GRDC 2020). However, in our field and pot experiments, we found poor control of *C. virgata* by this herbicide. We hypothesized that populations of *C. virgata* may differ in imazapic tolerance. Therefore, in this study, our interest was to access the tolerance levels of four populations of *C. virgata* at different doses of imazapic. In this case, 20 seeds of four populations of *C. virgata* (FTR3, FTR8, FTR9 and FTR11) were sown in pots (20 cm diameter and 18 cm height) filled with potting mix (Centenary Landscape, Australia). The populations of FTR were collected in 2017 from growers’ fields. The GPS coordinates of FTR3, FTR8, FTR9 and FTR11 were −26.8264, 150.5802; −28.0041, 148.4100; −28.0454, 148.3158; and −27.2935, 151.1283, respectively. The experimental design was a factorial randomized complete block design with three replicates where the first factor was populations and the second factor was imazapic doses [0*x* (no herbicide; control), 0.25*x*, 0.5*x*, 1*x*, 2*x* and 4*x*]. The 1*x* dose was the recommended dose (48 g ai ha^−1^) for imazapic. The study was started on 8 December 2020 and a similar procedure was used for herbicide spray as followed in Experiment 2. Plant survival was assessed 28 DAT (5 January 2021), and aboveground biomass was harvested, dried for 72 h at 70 °C and weighed. 

### 2.4. Statistical Analyses

Data of Experiment 1 and Experiment 2 were subjected to analysis of variance (ANOVA) using the software CPCS1. Treatment means were separated using Fisher’s protected least significant differences (LSD) at *p* ≤ 0.05. In Experiment 2, both runs were found significantly different, therefore, data of both runs were presented separately.

For Experiment 3, non-linear regression analysis was used to assess the survival percentage or biomass and imazapic doses. These data were explained with a functional three-parameter sigmoid model using SigmaPlot 14.0 (Systat Software, San Jose, CA, USA):

*G* (%) = *G_max_*/[1 + (*x*/*x*_50_) *^Grate^*] (1)

where *G* is the plant mortality % or the % reduction in weed biomass at imazapic dose *x*, *Gmax* is the maximum mortality % or the % reduction in biomass, *x*_50_ is the imazapic dose for 50% mortality or % reduction in biomass and *Grate* indicates the slope. 

## 3. Results

### 3.1. Experiment 1. Field Study

Lower emergence of mungbean was observed with flumioxazin (7 plants m^−2^) and isoxaflutole (3 plants m^−2^) treatments compared with a nontreated control (14 plants m^−2^) (Table 1). *C. virgata* density was lower in the plots treated with dimethenamid, isoxaflutole, pendimethalin, prosulfocarb + S-metolachlor, pyroxasulfone, S-metolachlor and trifluralin, respectively, compared with the nontreated control (Table 2). A similar trend was noticed for *C. virgata* biomass (Table 2). 

Weed biomass was reduced by 91%, 58%, 92%, 78%, 94%, 88% and 89% in plots treated with dimethenamid, isoxaflutole, pendimethalin, prosulfocarb + S-metolachlor, pyroxasulfone, S-metolachlor and trifluralin, respectively, compared with the nontreated control (Table 2). Imazapic, metribuzin and terbuthylazine were found to be not effective in controlling *C. virgata.*

*Chloris virgata* seed production was highest (64,500 seeds m^−2^) in the nontreated control plot and it was similar with the treatments metribuzin and terbuthylazine (Table 2). Seed production of *C. virgata* was reduced by 97%, 97%, 95%, 93%, 93%, 90% and 87% in plots treated with dimethenamid, trifluralin, S-metolachlor, pendimethalin, prosulfocarb + S-metolachlor, isoxaflutole and pyroxasulfone, respectively, compared with nontreated control.

Grain yield of mungbean was lowest in nontreated control (250 kg ha^−1^) and was similar in the plots treated with diuron, flumioxazin, isoxaflutole, metribuzin and terbuthylazine (Table 1). Grain yield was found to be the highest in the plots treated with pyroxasulfone (900 kg ha^−1^), and this yield was similar in the plots treated with dimethenamid, pendimethalin, prosulfocarb + S-metolachlor and S-metolachlor.

### 3.2. Experiment 2. Pot Study (PRE Herbicides)

In this pot study, the trend of *C. virgata* survival rate (%) and biomass in different herbicide treatments was similar between both experimental runs (Table 3). Dimethenamid, pendimethalin and S-metolachlor provided 100% control of *C. virgata.* (Table 3). The survival rate of *C. virgata* with an application of prosulfocarb + S-metolachlor, pyroxasulfone and trifluralin was less than 20%. A similar trend was noticed for biomass production (Table 3). 

The biomass of *C. virgata* did not reduce with the application of diuron, imazethapyr and terbuthylazine compared with nontreated control. Application of dimethenamid, pendimethalin and S-metolachlor provided 100% control of *C. virgata*. The biomass reduction with isoxaflutole, prosulfocarb + S-metolachlor and trifluralin was more than 80% compared with nontreated control.

### 3.3. Experiment 3. Pot Study (Imazapic Doses)

**Figure 1:**
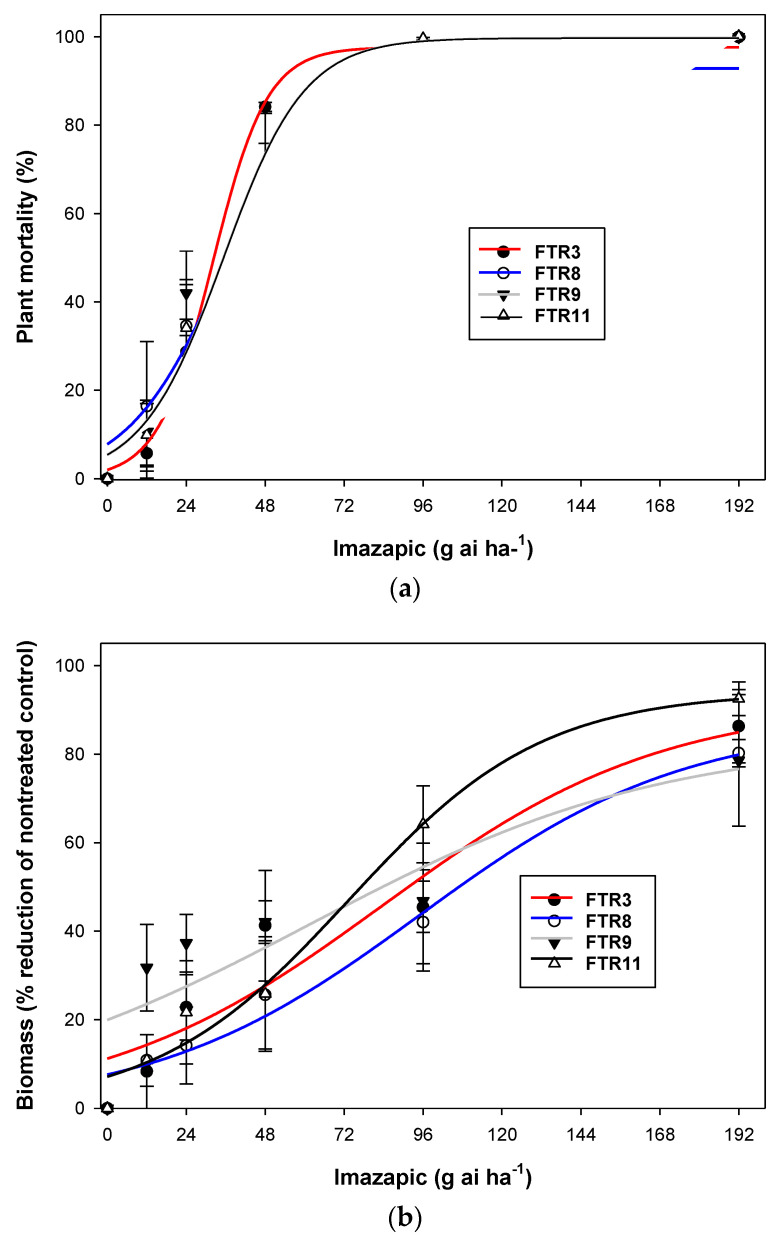
Plant mortality (**a**), and biomass (**b**) of *Chloris virgata* populations at different doses of imazapic (Experiment 3. Pot study). Error bars indicate the standard error of means. The curves for imazapic doses and populations FTR9 and FTR11 are superimposed; therefore, it is difficult to differentiate in the (**a**). Similarly symbols for each population at the highest dose are superimposed in (**a**).

For the dose-response curve, a three-parametric sigmoid model was fitted (Figure 1a,b) and parameter estimates have been provided in Table 4. In the dose-response study of imazapic, the plant mortality % of FTR3, FTR8, FTR9 and FTR11 was 85%, 66%, 74% and 74%, respectively, at the recommended dose of imazapic (48 g ai ha^−1^) (Figure 1). At the same dose, the % reduction in biomass of FTR3, FTR8, FTR9 and FTR11 was 28%, 21%, 36% and 28%. The imazapic dose required to reduce 50% biomass of FTR3, FTR8, FTR9 and FTR11 was 84, 95, 62 and 73 g ai ha^−1^, respectively (Figure 1). This study demonstrated that imazapic even at a higher dose (96 g ai ha^−1^) did not control the FTR8 population effectively (~44% reduction in biomass). Therefore, population FTR8 was found to be relatively tolerant to imazapic compared to other populations and required a relatively higher dose for weed suppression.

## 4. Discussion

Under the mungbean crop, ACCase-inhibitor herbicides are currently used for *C. virgata* control [6]. Even though ACCase-inhibitor herbicides currently provide effective control of *C. virgata*, this solution could be short-lived as the development of resistance in weeds against ACCase-inhibitor herbicides occurs quickly [8,15,20]. 

This study suggests that metribuzin and terbuthylazine provide poor control of *C. virgata*, and seed rains of survived plants can cause reinfestation of this weed in the paddocks. Seeds of *C. virgata* can be blown into paddocks from adjacent infested paddocks, as seeds are very light in weight and seeds may be deposited in the paddock via livestock, machinery or flood water. 

Our pot and field studies revealed that imazethapyr and imazapic provided moderate control of *C. virgata* and grain yield of mungbean did not improve to the extent as improved for pyroxasulfone and dimethenamid applications. Pendimethalin, trifluralin, imazethapyr and imazapic are recommended for general weed control in mungbean [21].Imazethapyr and imazapic herbicides are acetolactate synthase (ALS) and acetohydroxyacid synthase (AHAS) inhibitors that caused inhibition of branched-chain amino acids [22]. Imazethapyr and imazapic are imidazolinone herbicides and have been registered for use in pulses [23,24]. A previous study suggested that imazethapyr at 100 g ai ha^−1^ PRE reduced grain and straw yield of mungbean and did not control weeds effectively [25]. In the current study, we found poor control of *C. virgata* with imazethapyr; however, imazethapyr is a registered herbicide for grass weed control in Australia [15]. In Australia, imazapic is not a registered herbicide for weed control in mungbean. This study suggests that imazethapyr and imazapic did not cause crop toxicity to mungbean, however, they also did not control *C. virgata* effectively.

Pendimethalin and trifluralin are microtubule assembly inhibitors and inhibit cell wall formation [26]. Pendimethalin and trifluralin inhibit microtubule polymerization, which interferes with cell division and cell wall formation in sensitive weed seedlings. Secondary root formation in weeds is halted, resulting in reduced water and nutrient absorption from the soil [26,27]. Pendimethalin and trifluralin are registered herbicides for weed control in mungbean. Our results revealed that pendimethalin and trifluralin could effectively control *C. virgata*, and that timely application of these herbicides may improve mungbean yield and reduce the seed production of *C. virgata* for further reinfestation. Previous studies suggested that pendimethalin 500–1000 g ai ha^−1^ resulted in an improved yield of mungbean due to effective weed control [17,18,25,28,29]. 

Crop phytotoxicity was observed in the field trial with the application of isoxaflutole and flumioxazin (visual observations). Isoxaflutole provided moderate control of *C. virgata*; however, it caused toxicity to mungbean. Therefore, the grain yield of mungbean did not improve in the isoxaflutole-treated plots. 

Flumioxazin inhibits protoporphyrinogen oxidase (PPO), an enzyme of chlorophyll and heme biosynthesis. Lipids and proteins are attacked by this herbicide and oxidized resulting in loss of chlorophyll and carotenoids [30]. However, the loss of chlorophyll and carotenoids depends on weed/crop species and herbicide rate used. Our field study suggests that flumioxazin 90 g ai ha^−1^ could cause crop injury in mungbean and reduce yield. Previous studies suggested that flumioxazin applied PRE at 142 g ai ha^−1^ caused up to 84% adzuki bean (*Vigna angularis* Willd.) injury, which resulted in >50% grain yield loss [31]. In our field study, flumioxazin provided good control *C. virgata*; however, it resulted in reduced grain yield compared with pyroxasulfone (highest yield treatment). 

Isoxaflutole causes inhibition of 4-hydroxyphenyl-pyruvate dioxygenase (4-HPPD) that results in bleaching in plants. In Australia, isoxaflutole 70 g ai ha^−1^ is registered for weed control in sugarcane (*Saccharum officinarum* L.) and chickpea (*Cicer arietinum* L.). However, under a non-cropped situation, it is also registered for *C. virgata* control. In this study, isoxaflutole was found to be effective against *C. virgata*; however, it also toxic to the mungbean, resulting in reduced yield. A previous study revealed that bleaching and necrosis occurred in isoxaflutole-treated mungbean leaves and it caused reduced shoot growth [32]. It was reported that isoxaflutole caused inhibition of HPPD activity that resulted in inhibition of carotenoid biosynthesis [33]. These results, including the present study, suggest that isoxaflutole is not a safe herbicide for *C. virgata* control in mungbean.

Dimethenamid-P, S-metolachlor and, pyroxasulfone are chloroacetamides, very long-chain fatty acid (VLCFA) inhibitor herbicides and are known for excellent control of grass weeds [24]. These compounds affect weeds before emergence but do not inhibit seed germination. Dimethenamid, S-metolachlor and pyroxasulfone are not registered for mungbean in Australia. We did not observe any crop toxicity in mungbean with dimethenamid-P, S-metolachlor and pyroxasulfone, and they provided excellent control of *C. virgata*. Our study suggests that these three herbicides, dimethenamid-P, S-metolachlor and pyroxasulfone, can effectively kill *C. virgata* and reduce its seed production and may help improve mungbean yield. 

S-metolachlor has been registered for *C. virgata* control in Australia but not specifically in the mungbean crop [15]. Previous studies suggested that closely related species of mungbean, such as adzuki, have the tolerance to S-metolachlor and dimethenamid-P. Another study reported negligible (≤2%) injury for PRE dimethenamid-P at up to 440 g ai ha^−1^ [34]. Crop tolerance to dimethenamid-P is influenced by soil type, where a greater cation exchange capacity has the potential to increase crop injury [35]. However, we did not observe any crop toxicity of dimethenamid-P in this study. A previous study suggested that S-metolachlor at 1400 g ai ha^−1^ delayed maturity when applied at the unifoliate growth stage but did not reduce grain yield of kidneybean (*Phaseolus vulgaris* L.) [36]. 

Dimethenamid-P or S-metolachlor PRE, at 1300 or 2800 g ai ha^−1^, respectively, injured *kidneybean* cultivars, but grain yield was not reduced in a cultivar tolerance field study [36]. In a planting date study, dimethenamid-P PRE at 2300 g ai ha^−1^ reduced leaf area and delayed maturity of kidney beans compared with the nontreated control when pooled over five planting dates and cultivars [36]. A previous study confirmed that wheat (*Triticum aestivum* L.) and grain legumes have a higher level of tolerance against pyroxasulfone than other cereals and canola (*Brassica napus* L.) [37]. These authors also confirmed that pyroxasulfone was a good option for controlling *Lolium rigidum* Gaudin.

Terbuthylazine, linuron, metribuzin, atrazine and diuron are photosystem II inhibitors and caused inhibition of photosynthesis at photosystem II [38]. These herbicides are not registered for weed control in mungbean in Australia. This study suggests that although these herbicides did not cause crop toxicity in mungbean, they did not control *C. virgata* also.

Prosulfocarb + S-metolachlor and S-metolachlor are absorbed by the roots and shoots (coleoptile) of germinating seedlings and caused inhibition of growth in the meristematic region. In Australia, these herbicides are used for weed control in barley (*Hordeum vulgare* L.), chickpea, fababeans (*Vicia faba* L.) and wheat (APVMA 2021, https://apvma.gov.au/; accessed on 5 June 2021). In the current study, both herbicides did not cause crop toxicity in mungbean and provided effective control of *C. virgata*. This study suggests that both herbicides can be used for *C. virgata* control in mungbean.

This study suggests that *C. virgata* is a troublesome weed of mungbean in eastern Australia. Once *C. virgata* is established in the field, controlling it becomes a difficult task and a costly affair. This study also revealed that the seed production of *C. virgata* is remarkably high and under noncontrolled conditions in mungbean, it can produce more than 60,000 seeds m^−2^. Therefore, it is necessary to control *C. virgata* plants before they set seeds. 

In Australia, the residual herbicide flumioxazin has been recommended for *C. virgata* control. However, our study revealed that herbicides that are not registered for mungbean provided better control of *C. virgata* than flumioxazin. In this study, PRE herbicides such as dimethenamid-P, pyroxasulfone, prosulfocarb, pendimethalin and trifluralin provided effective control of *C. virgata* and helped in improving the yield of mungbean by reducing the infestation of the weed. 

In conclusion, PRE application of dimethenamid-P, S-metolachlor, pyroxasulfone, pendimethalin and trifluralin at the tested rate (Table 1) provided effective control (89%) of *C. virgata* and reduced (>89%) its seed production. Isoxaflutole caused injury to the mungbean crop and resulted in lower yield compared with dimethenamid-P, S-metolachlor, pyroxasulfone, pendimethalin and trifluralin. Flumioxazin, although currently being labelled for *C. virgata* control, did not provide excellent control of *C. virgata* at the tested rate. Metribuzin and terbuthylazine were found to be safe for the mungbean crop, but these herbicides did not control *C. virgata*. Imazapic and imazethapyr did not control *C. virgata*. Further studies are needed to determine the efficacy of herbicides such as dimethenamid-P, S-metolachlor and pyroxasulfone to control other troublesome weeds in mungbean.

## Figures and Tables

**Table 1:** Effect of herbicides on emergence, crop phytotoxicity score and grain yield of mungbean (Field study).

Herbicides	Dose (g ai ha^−1^)	Emergence (Plants per Meter Row Length)	Crop Phytotoxicity Score	Grain Yield (kg ha^−1^)
Nontreated control	-	14b	0	247a
Dimethenamid-P	720	9b	20	805cd
Diuron	747	11b	10	462ab
Flumioxazin	90	7ab	40	590b
Imazapic	48	14b	10	483b
Imazethapyr	70	13b	10	534b
Isoxaflutole	75	3a	95	349ab
Linuron	1125	12b	10	448ab
Metribuzin	360	9b	15	201a
Pendimethalin	1000	13b	10	807cd
Prosulfocarb + S-metolachlor	2300	10b	10	751cd
Pyroxasulfone	100	11b	15	900d
S-metolachlor	1440	13b	10	677cd
Terbuthylazine	752	10b	10	398ab
Trifluralin	600	10b	15	650bc
LSD (0.05)	-	5	-	226

LSD; Least significant differences; values in the table are mean of three replications; Means followed by similar letters are not significantly different (*p* < 0.05)-using Duncan test.

**Table 2:** Effect of herbicides on density, biomass and seed production of *Chloris virgata* (Experiment 1. Field study).

Herbicides	Dose (g ai ha^−1^)	*C. virgata* Density (Plants m^−2^)	*C. virgata* Biomass (g m^−2^)	*C. virgata* Seed (no. m^−2^)
Nontreated	-	8.0bc	350c	64,480c
Dimethenamid-P	720	2.7a	30a	2080a
Diuron	747	10.7c	152b	11,440ab
Flumioxazin	90	10.7c	74a	12,480ab
Imazapic	48	12.0c	241bc	19,760ab
Imazethapyr	70	8.0bc	181b	13,520ab
Isoxaflutole	75	2.7a	147b	6240ab
Linuron	1125	10.7c	179b	17,680ab
Metribuzin	360	10.7c	298bc	49,920bc
Pendimethalin	1000	4.0ab	29a	4160a
Prosulfocarb + S-metolachlor	2300	4.0ab	78a	8320ab
Pyroxasulfone	100	4.0ab	22a	4160a
S-metolachlor	1440	4.0ab	43a	3120a
Terbuthylazine	752	10.7c	321c	39,520bc
Trifluralin	600	4.0ab	37a	2080a
LSD (0.05)	-	5.2	120	34,848

LSD; Least significant differences; values in the table are the mean of three replications; Means followed by similar letters are not significantly different (*p* < 0.05)—using Duncan test.

**Table 3:** Effect of herbicides on survival % and biomass of *Chloris virgata* (Experiment 2. pot study).

Herbicides	Dose (g ai ha^−1^)	Survival % (Run 1)	Survival % (Run 2)	Biomass (g per Pot) (Run 1)	*C. virgata* Biomass (g per Pot) (Run 2)
Nontreated control	-	35b	67d	6.8c	3.8c
Atrazine	2700	35b	52cd	4.2b	1.3b
Dimethenamid-P	720	0a	0a	0.0a	0.0a
Diuron	747	35b	58cd	5.6c	3.8c
Imazapic	48	28b	47b	3.6b	1.9b
Imazethapyr	70	32b	62c	5.6c	3.6c
Isoxaflutole	75	8a	40b	0.4a	1.2b
Metribuzin	360	30b	57c	5.9c	1.2b
Pendimethalin	1000	0a	0a	0.0a	0.0a
Prosulfocarb + S-metolachlor	2300	7a	0a	0.1a	0.0a
Pyroxasulfone	100	2a	2a	0.02a	0.03a
S-metolachlor	1440	0a	0a	0.0a	0.0a
Terbuthylazine	752	35b	67d	6.6	3.8c
Triallate	800	27b	32b	4.14b	1.2b
Trifluralin	600	7a	5a	1.3a	0.04a
LSD (0.05)	-	12	18	1.3	0.9

LSD; Least significant differences; values in the table are the mean of six replications; Means followed by similar letters are not significantly different (*p* < 0.05)-using Duncan test.

**Table 4:** Estimates of regression parameters for plant mortality (%) and biomass reduction (%) of four populations of *Chloris virgata* in relation to imazapic doses.

Population	*G*_max_	*G*_rate_	*x*_50_	*R* ^2^
Plant mortality (%)				
FTR3	6420 (102)	−117 (88)	−531 (135)	0.95
FTR8	108 (54)	−78 (42)	74 (9)	0.94
FTR9	19,516 (187)	−152 (67)	−886 (184)	0.73
FTR11	108 (42)	−47(23)	56 (44)	0.96
Biomass reduction (%)				
FTR3	4 (0.2)	−8 (1.3)	33 (2)	0.99
FTR8	5 (1.4)	−31 (9)	29 (20)	0.99
FTR9	4 (0)	−8 (0)	33 (0)	0.99
FTR11	4 (0.3)	−14 (2)	34 (3)	0.99

Values in praentheses indicate standard errors (±).

## Data Availability

All relevant data are within the manuscript.

## References

[1] Clarry S. The Rise and Rise of Mungbeans. GroundCover Issue 125—Pulse Breeding Advances. https://grdc.com.au/Media-Centre/Ground-Cover-Supplements/Ground-CoverIssue-125--Pulse-breeding-advances/The-rise-and-rise-of-mungbeans2016.

[2] Rachaputi R.C.N., Sands D., McKenzie K., Agius P., Lehane J., Seyoum S. (2019). Eco-physiological drivers influencing mungbean *Vigna radiata* (L.) Wilczek productivity in subtropical Australia. Field Crop. Res..

[3] Chauhan B.S., Florentine S.K., Ferguson J.C., Chechetto R.G. (2017). Implications of narrow crop row spacing in managing weeds in mungbean (*Vigna radiata*). Crop. Prot..

[4] Yadav S.K., Bhan V.M., Singh S.P. (1983). Crop-weed competition studies in mung beans (*Vigna radiata*). Exp. Agric..

[5] Davidson B., Cook T., Chauhan B.S. (2019). Alternative options to glyphosate for control of large *Echinochloa colona* and *Chloris virgata* plants in cropping fallows. Plants.

[6] Desai H.S., Thompson M., Chauhan B.S. (2020). Target-site resistance to glyphosate in *Chloris virgata* biotypes and alternative herbicide options for its control. Agronomy.

[7] Fernando N., Humphries T., Florentine S.K., Chauhan B.S. (2016). Factors affecting seed germination of feather fingergrass (*Chloris virgata*). Weed Sci..

[8] Ngo T.D., Krishnan M., Boutsalis P., Gill G., Preston C. (2018). Target-site mutations conferring resistance to glyphosate in feathertop Rhodes grass (*Chloris virgata*) populations in Australia. Pest. Manag. Sci..

[9] Widderick M., McLean A. (2018). Optimal intervals differ for double knock application of paraquat after glyphosate or haloxyfop for improved control of *Echinochloa colona*, *Chloris virgata* and *Chloris truncata*. Crop. Prot..

[10] Llewellyn R.S., Ronning D., Ouzman J., Walker S., Mayfield A., Clarke M. (2016). Impact of Weeds on Australian Grain Production: The Cost of Weeds to Australian Grain Growers and the Adoption of Weed Management and Tillage Practices.

[11] Ngo T.D., Boutsalis P., Preston C., Gill G. (2017). Growth, development, and seed biology of feather fingergrass (*Chloris virgata*) in Southern Australia. Weed Sci..

[12] Manalil S., Mobli A., Chauhan B.S. (2020). Competitiveness of windmill grass (*Chloris truncata*) and feathertop Rhodes grass (*Chloris virgata*) in mungbean (*Vigna radiata*). Crop. Pasture Sci..

[13] Werth J., Boucher L., Thornby D., Walker S., Charles G. (2013). Changes in weed species since the introduction of glyphosate-resistant cotton. Crop. Pasture Sci..

[14] Manalil S., Werth J., Jackson R., Chauhan B.S., Preston C. (2017). An assessment of weed flora 14 years after the introduction of glyphosate-tolerant cotton in Australia. Crop. Pasture Sci..

[15] Grain Research and Development Corporation. https://grdc.com.au/resources-and-publications/all-publications/publications/2020/integrated-weed-management-of-feathertop-rhodes-grass?utm_medium=short_url&utm_content=Integrated%20Weed%20Management%20of%20Feathertop%20Rhodes%20Grass&utm_source=website&utm_term=North.

[16] Kaur S., Kaur T., Bhullar M.S. (2016). Imidazolinone herbicides for weed control in greengram. Indian J. Weed Sci..

[17] Khairnar C.B., Goud V.V., Sethi H.N. (2014). Pre- and post-emergence herbicides for weed management in mungbean. Indian J. Weed Sci..

[18] Patel B.D., Chaudhari D.D., Patel V.J., Patel R.B. (2016). Pre- and post-emergence herbicides for weed control in greengram and their residual effect on succeeding crops. Indian J. Weed Sci..

[19] Mahajan G., Walsh M., Chauhan B.S. (2020). Junglerice (*Echinochloa colona*) and feather fingergrass (*Chloris virgata*) seed production and retention at sorghum maturity. Weed Technol..

[20] Heap I. International Survey of Herbicide Resistant Weeds. www.weedscience.org.

[21] Grain Research and Development Corporation. https://grdc.com.au/__data/assets/pdf_file/0014/315311/GRDC-GrowNotes-Mungbeans-Northern.pdf.

[22] LaRossa R.A., Schloss J.V. (1984). The sulfonylurea herbicide sulfometuron methyl is an extremely potent and selective inhibitor of acetolactate synthase in Salmonella typhimurium. J. Biol. Chem..

[23] BASF (2011). Pursuit Herbicide.

[24] Mallory-Smith C.A., Retzinger E.J. (2003). Revised classification of herbicides by site of action for weed resistance management strategies. Weed Technol..

[25] Ali S., Patel J.C., Desai L.J., Singh J. (2011). Effect of herbicides on weeds and yield of rainy season greengram (*Vigna radiata* L. Wilczek). Legume Res..

[26] Naylor E.L. (2008). Weed Management Handbook.

[27] Senseman S.A., Armbrust K. (2007). Herbicide Handbook.

[28] Buttar G.S., Aulakh C.S., Mehra S.P. (2006). Chemical weed control in mungbean (*Vigna radiata* L.)—Farmer’s participatory approach. Indian J. Weed Sci..

[29] Mirjha P.R., Prasad S.K., Patel S., Baghel P., Monesh S. (2013). Effect of chemical weed control on weed indices in kharif mungbean (*Vigna radiata* L. Wilczek). Environ. Ecol..

[30] Duke S.O., Lydon J., José M.B., Sherman T.D., Lehnen L.P., Matsumoto H. (1991). Protoporphyrinogen oxidase-inhibiting herbicides. Weed Sci..

[31] Soltani N., Nurse R.E., Shropshire C., Sikkema P.H. (2015). Tolerance of adzuki bean to pre-emergence herbicides. Can. J. Plant Sci..

[32] Kaushik S. (2006). Phytotoxicity of selected herbicides to mung bean (*Phaseolus aureus* Roxb.). Environ. Exp. Bot..

[33] Bhowmik P.C., Swarcewicz M.K., Mitra S. Plant bioassay for isoxaflutole in soil. Proceedings of the 18th Asian Pacific Weed Science Society Conference.

[34] Soltani N., Shropshire C., Sikkema P.H. (2014). Sensitivity of dry bean to dimethenamid-p, saflufenacil and dimethenamid-p/saflufenacil. Am. J. Plant Sci..

[35] Macleod I.J., Frost P.R. Dimethenamid-P—A new selective herbicide for Australian horticulture. Proceedings of the 13th Australian Weeds Conference.

[36] Poling K.W., Renner K.A., Penner D. (2009). Dry edible bean class and cultivar response to dimethenamid and metolachlor. Weed Technol..

[37] Walsh M.J., Fowler T.M., Crowe B., Ambe T., Powles S.B. (2011). The potential for pyroxasulfone to selectively control resistant and susceptible rigid ryegrass (*Lolium rigidum*) biotypes in Australian grain crop production systems. Weed Technol..

[38] Devine M., Duke S.O., Fedtke C. (1993). Physiology of Herbicide Action.

